# Suppression of NAD(P)H-quinone oxidoreductase 1 enhanced the susceptibility of cholangiocarcinoma cells to chemotherapeutic agents

**DOI:** 10.1186/1756-9966-33-11

**Published:** 2014-01-24

**Authors:** Ponsilp Zeekpudsa, Veerapol Kukongviriyapan, Laddawan Senggunprai, Banchob Sripa, Auemduan Prawan

**Affiliations:** 1 Department of Pharmacology, Faculty of Medicine, Khon Kaen University, Khon Kaen 40002, Thailand; 2 Department of Pathology, Faculty of Medicine, Khon Kaen University, Khon Kaen, Thailand; 3 Liver Fluke and Cholangiocarcinoma Research Center, Khon Kaen University, Khon Kaen, Thailand

**Keywords:** NAD(P)H-quinone oxidoreductase 1 (NQO1), Cholangiocarcinoma (CCA), 5-fluorouracil (5-FU), Doxorubicin (Doxo), Gemcitabine (Gem), p53

## Abstract

**Background:**

Cholangiocarcinoma (CCA) is highly resistant to most of the known chemotherapeutic treatments. NAD(P)H-quinone oxidoreductase 1 (NQO1) is an antioxidant/detoxifying enzyme recently recognized as an important contributor to chemoresistance in some human cancers. However, the contribution of NQO1 to chemotherapy resistance in CCA is unknown.

**Methods:**

Two CCA cell lines, KKU-100 and KKU-M214, with high and low NQO1 expression levels, respectively, were used to evaluate the sensitivity to chemotherapeutic agents; 5-fluorouracil (5-FU), doxorubicin (Doxo), and gemcitabine (Gem). NQO1 and/or p53 expression in KKU-100 cells were knocked down by siRNA. NQO1 was over-expressed in KKU-M214 cells by transfection with pCMV6-XL5-NQO1 expression vector. CCA cells with modulated NQO1 and/or p53 expression were treated with chemotherapeutic agents, and the cytotoxicity was assessed by SRB assay. The mechanism of enhanced chemosensitivity was evaluated by Western blot analysis.

**Results:**

When NQO1 was knocked down, KKU-100 cells became more susceptible to all chemotherapeutic agents. Conversely, with over-expression of NQO1 made KKU-M214 cells more resistant to chemotherapeutic agents. Western blot analysis suggested that enhanced chemosensitivity was probably due to the activation of p53-mediated cell death. Enhanced susceptibility to chemotherapeutic agents by NQO1 silencing was abolished by knockdown of p53.

**Conclusions:**

These results suggest that inhibition of NQO1 could enhance the susceptibility of CCA to an array of chemotherapeutic agents.

## Background

Cholangiocarcinoma (CCA) is a malignant cancer arising from neoplastic transformation of cholangiocytes, the epithelial cells lining of intrahepatic and extrahepatic bile duct [1,2]. The incidence of CCA is extremely high in northeastern Thailand [3,4]. The most important risk factor is the liver fluke (*Opisthorchis viverrini*) infection [5,6]. Several lines of studies have shown that the incidence and mortality rates of intrahepatic CCA are increasing worldwide [2,7]. The prognosis is generally poor because most patients present at advanced disease and early diagnosis is difficult [7]. Curative surgical resection is considered the most effective treatment, but most cases are inoperable at the time of diagnosis [7]. Unfortunately, chemotherapeutic agents are modestly effective on CCA and drug resistance is the major obstacle in the treatment. Multiple mechanisms are assumed to be involved in drug resistance; *e.g.*, alteration of drug metabolizing enzymes, efflux transporters, cytoprotective enzymes or derangement of intracellular signaling system [8]. It is an urgent need to search for novel treatments for CCA.

NAD(P)H-quinone oxidoreductase 1 (NQO1 or DT-diaphorase, EC 1.6.99.2) is a drug metabolizing enzyme. Its over-expression has been observed in many cancers of the liver, thyroid, breast, colon, and pancreas [9,10]. NQO1 is a flavoprotein mainly expressed in cytosol, catalyzing an obligate two-electron reduction of a broad range of substrates, particularly quinines, quinone-imines, nitro and azo compounds as the most efficient substrates [11-15]. NQO1 has several functions including xenobiotic detoxification, superoxide scavenging, and modulation of p53 proteasomal degradation [12]. Chronic inflammation suppresses NQO1 expression [16] and may increase susceptibility to cell injury. Increasing number of evidences suggest that up-regulation of NQO1 at the early process of carcinogenesis may provide cancer cells a growth advantage [17,18]. Since NQO1 is also an antioxidant enzyme, it may protect cancer cells by removing free radicals and making cells more resistant to anticancer agents, particularly to oxidative stress inducers.

Recently, a role of NQO1 in cancer chemotherapy has been demonstrated by several groups. Inhibition of NQO1 by a pharmacological inhibitor, dicoumarol, suppressed urogenital and pancreatic cancer cell growth and also potentiated cytotoxicity of cisplatin and doxorubicin [19,20]. Significant association was observed between high NQO1 expression in CCA tissue and short survival [21]. We have recently demonstrated that dicoumarol potentiated gemcitabine-induced cytotoxicity on CCA cells with high NQO1 activity [22]. The chemosensitizing effect was associated with oxidative stress and induction of p53 protein [20]. However, dicoumarol could exert several effects apart from inhibition of NQO1, such as suppression of JNK and NF-κB pathways, and potentiation of apoptosis induced by TNF-α in HeLa cells [23]. The exact mechanism of the chemosensitizing effect conferred by suppression of NQO1 still remains unclear. The importance of NQO1 on modulation of p53 is also conflicting [22,24].

In the present study, we validate the role of NQO1 in cytoprotection, and then demonstrate that suppression of NQO1 potentiates antitumor activity of chemotherapeutic agents. These results suggest the crucial role of NQO1 in cancer cells. NQO1 may be a potential target molecule to enhance the susceptibility of tumor cells to chemotherapeutic agents.

## Methods

### Human cell line cultures and chemotherapeutic agents

Two human CCA cell lines, KKU-100 and KKU-M214, were developed from tumor tissues of CCA patients at the Srinagarind Hospital, Faculty of Medicine, Khon Kaen University. Liver Chang cells and normal bile duct epithelial cells, MMNK1, were also used in this study. CCA cells and normal cells were routinely cultured in Ham’s F12 media, supplemented with 4 mmol/L L-glutamine, 12.5 mmol/L N-2-hydroxyethylpiperazine-N’-2-ethanesulfonic acid (HEPES), at pH 7.4, 100 U/mL penicillin, 100 μg/mL streptomycin sulfate, and 10% fetal bovine serum (FBS) in a humidified atmosphere containing 5% CO_2_ at 37°C. The media was renewed every 2–3 days. After the cells became confluent, cells were trypsinized with 0.25% trypsin-EDTA and subcultured in the same media. Some aliquots of cells were transferred to freezing medium containing 10% DMSO and stored at -80°C for subsequent use.

Chemotherapeutic agents were selected on the basis of the frequent usage for CCA, gastrointestinal tract cancers and solid tumors. These included 5-fluorouracil (5-FU) dissolved in DMSO (100 mM), doxorubicin HCl (Boryung Pharm, Seoul, South Korea: Doxo) dissolved in DMSO (100 mM), and gemcitabine (Gemzar, Eli Lilly, IN, USA: Gem) dissolved in phosphate-buffered saline (PBS: 137 mM NaCl, 2.7 mM KCl, 10 mM Na_2_HPO_4_, 2 mM KH_2_PO_4_, pH 7.4). They were added to the culture media without FBS to make final concentrations indicated in the “Results” section and incubated for a designated period of time.

### Transient transfection of NQO1 and/or p53 small interfering RNA (NQO1 and/or p53 siRNA)

Pre-designed NQO1 siRNA (siGENOME SMARTpool siRNA #M-005133-02-0020), p53 siRNA (siGENOME SMARTpool siRNA #M-003329-03-0005), and control siRNA (siGENOME non-targeting siRNA #D-001210-02-20) were purchased from Thermo Scientific. In this study, NQO1 siRNA and p53 siRNA were the pooled siRNAs, each is composed of four different sequences of siRNA, targeting for NQO1 and p53, respectively. For transfection of the siRNA, 1.5×10^5^ KKU-100 cells were plated in 6-well plates and grown in Ham’s F12 medium supplemented with FBS, without antibiotics. The cells were transfected with 50 or 100 pmole of the siRNA for 6 hr using 0.4 or 2 μL of Lipofectamine™ 2000 reagent (Invitrogen, Calsbad, CA, USA) in 500 μL of Ham’s F12 medium without FBS and antibiotics. After transfection, the cells were added with 1.5 mL of Ham’s F12 medium supplemented with FBS, without antibiotics, and incubated further for 24-48 hr. The efficiency of the NQO1 knockdown by transient transfection was determined by gene expression with reverse transcription real-time polymerase chain reaction (RT-qPCR) using specific primers, NQO1 activity assay, and Western blotting analysis.

For cytotoxicity assay, CCA cells were seeded onto 96-well cultured plates with FBS, without antibiotics at a density of 5 × 10^3^ cells/well for an overnight. The cells were transfected with 3 pmole of the siRNA for 6 hr using 0.06 μL of Lipofectamine™ 2000 reagent in 100 μL of Ham’s F12 medium without FBS and antibiotics. After 6 hr, the cells were added 100 μL of Ham’s F12 medium supplemented with FBS, without antibiotics, and incubated for 48 hr. The cells were then incubated with chemotherapeutic agents in serum free medium for additional 24 hr.

### Transfection of NQO1 vector into CCA cells

A plasmid encoding human wild-type NQO1 in pCMV6-XL5 (4,707 bp) was purchased from Origene Technologies (#SC119599; Rockville, MD). The insert cDNA (1,120 bp) contained the complete NQO1 coding sequence (NM_000903.2). For transfection of the pCMV6-XL5-NQO1 or pCMV6-XL5, as a negative control vector, KKU-M214 at a density of 5×10^5^ cells were plated in 6-well plates and grown overnight. At 70-80% confluent condition, cells were transfected with 2.5 μg of pCMV6-XL5-NQO1 or pCMV6-XL5 for 24 hr using Lipofectamine^®^ LTX and Plus™ reagent (Invitrogen) protocol as directed by the manufacturer in 2 mL of Ham’s F12 medium without FBS and antibiotics. Then the cells were collected for Western blot analysis and enzymatic assay. The empty vector control was prepared by cutting the NQO1 insert site from pCMV6-XL5-NQO1 plasmid at the *Eco*RI and *Xbal*I site. The bearing vector was ligated with oligonuclotide (non-coding sequence) and cloned into *E. coli* (JM109). The empty vector control was purified and the presence of vector was confirmed by restriction digestion and run it on 2% agarose gel.

For cytotoxicity assay, KKU-M214 cells were seeded onto 96-well cultured plates at a density of 7.5 × 10^3^ cells/well for an overnight, the cells were transfected with 100 ng of pCMV6-XL5-NQO1 or pCMV6-XL5 using Lipofectamine^®^ LTX and Plus™ reagent for 24 hr. The cells were then incubated with chemotherapeutic agents in serum free medium for additional 24 hr (Doxo) or 48 hr (5-FU and Gem), since it was the optimal incubation time for each drug.

### NQO1 enzyme activity assay

NQO1 assay was performed according to the method described previously [20]. Cells were seeded at 7.5 × 10^3^ cells/well in flat-bottomed 96-well cultured plates overnight. After cells were cultured for the designated time, cells were lysed with 50 μL solution containing 0.8% digitonin and agitated on a shaker at room temperature for 10 min. Twenty-five microliter of 0.55% dicoumarol was added into culture wells designated as baseline activity, while the corresponding paired wells were added with distilled water (DW) designated as the test activity wells. After that, all wells were added with 200 μL of reaction mixture (the following stock solution was prepared for each set of assay: 7.5 mL of 0.5 M Tris–HCl (pH 7.4), 100 mg of bovine serum albumin (BSA), 1 mL of 1.5% Tween-20 solution, 0.1 mL of 7.5 mM FAD, 1 mL of 150 mM glucose-6-phosphate, 100 μL of 50 mM β-NADP, 275 unit of yeast glucose-6-phosphate dehydrogenase, 45 mg of MTT, and DW to a final volume of 150 mL and menadione (1 μL of 50 mM menadione dissolved in acetonitrile per milliliter of reaction mixture) was added just before the mixture is dispensed into the microtiter plates. A blue color developed and the plates were placed into a microplate reader with filter wavelength of 620 nm and readings were made at 0.5 min interval for about 10 min. The rate of increase of the optical readings with times represents the activity of the reaction. Using the extinction coefficient of MTT formazan of 11,300 M^-1^ cm^-1^ at 610 nm and correction for the light path of the microplate, NQO1 activity was expressed as nmol/min/mg protein.

### Cytotoxicity or SRB assay

Cytotoxicity testing is used to evaluate the effects of chemotherapeutic agents. In brief, CCA cells were seeded onto 96-well cultured plates at a density of 7.5 × 10^3^ cells/well overnight, then media was renewed with fresh media containing test compound and further incubated for the indicated times. Assay was performed at the endpoint of treatment to determine amount of protein remaining in each well. Media was discarded and replaced with 100 μL of ice-cold 10% trichloroacetic acid (TCA) and placed in 4°C for at least 1 hr. Then TCA was removed and wells were carefully rinsed with deionized (DI) water for 5 times. After 10 min of air drying, 50 μL of 0.4% sulforhodamine B (SRB) in 1% acetic acid was added for 30 min. Cells were rinsed 3–4 times with 1% acetic acid and air dried for 1 hr at room temperature. Finally, adhered cells were solubilized with 200 μL of 10 mM Tris base and plates were shaken for 20 min before absorbance reading with a microplate reader with filter wavelength of 540 nm.

### Real-time polymerase chain reaction (real-time PCR or qPCR)

CCA cells were seeded in 6-well plates at the density of 1.5×10^5^ cells/well. Total RNA was extracted from CCA cell lines using TRIzol^®^ reagent following the manufacturer’s instructions (Invitrogen). Total RNA was isolated using a previously described method [20]. Total RNA (1 μg) was reverse transcribed in a 20 μL reaction mixture, containing 0.5 μg of oligo(dT)15 primer, 20 U of RNasin^®^ ribonuclease inhibitor, and 200 U of ImProm-II™ reverse transcriptase in 1× PCR buffer, 3 mmol/L MgCl_2_, and 1 mmol/L dNTPs. The first-strand cDNA was synthesized at conditions of 42°C for 60 min. The reverse transcription products served as templates for real-time PCR. PCR amplification was performed using specific primers for the NQO1, wild type p53 and the internal control using β-actin. The primer sequences were as follows: 1) NQO1 (NM_000903.2): forward primer 5’-GGCAGAAGAGCACTGATCGTA-3’ and reverse primer 5’-TGATGGGATTGAAGTTCATGGC-3’; 2) wild type p53 (NM_005256778.1) [25]: forward primer 5’-ATGGAGGAGCCGCAGTCAGATCC-3’ and reverse primer 5’-TTCTGTCTTCCCGGACTGAGTCTGACT-3’; 3) β-actin: forward primer 5’-TGCCATCCTAAAAGCCAC-3’ and reverse primer 5’-TCAACTGGTCTCAAGTCAGTG-3’. The real-time fluorescence PCR, based on EvaGreen^®^ dye, was carried out in a final volume of 20 μL containing 1x SsoFast™ EvaGreen^®^ supermix (#172-5200; Bio-Rad, CA, USA), 0.5 μmol/L of each NQO1 or wild type p53, and 0.25 μmol/L of β-actin primer. Thermal cycling was performed for each gene in duplicate on cDNA samples in 96-well reaction plates using the ABI 7500 Sequence Detection system (Applied Biosystems). A negative control was also included in the experimental runs. The negative control was set up by substituting the template with DI water. Real-time PCR was conducted with the following cycling conditions: 95°C for 3 min, followed by 40 cycles of 95°C for 15 s and 60°C for 31 s. To verify the purity of the products, a melting curve analysis was performed after each run. Upon completion of 40 PCR amplification cycles, there was a dissociation step of ramping temperature from 60°C to 95°C steadily for 20 min, while the fluorescence signal was continually monitored for melting curve analysis. The concentration of PCR product was calculated on the basis of established standard curve derived from serial dilutions of the positive control for NQO1, wild type p53 and β-actin in the CCA cell lines.

### Western blot analysis

After treatment with chemotherapeutic agents, CCA cells were washed with PBS, collected, and lysed at 4°C with 1x cell lysis buffer with 1 mmol/L dithiothreitol and 0.1 mmol/L phenylmethylsulfonyl fluoride (PMSF) with vigorous shaking. After centrifugation at 12,000 *g* for 30 min, supernatant was collected and stored at -80°C until use. Thirty microgram of the protein samples were mixed with 5x loading-dye buffer, heated at 90°C for 10 min, and proteins were then separated by electrophoresis in 10% SDS-polyacrylamide gel. Proteins were transferred to polyvinylidene difluoride (PVDF) membranes at 180 mA for 1 hr. The PVDF membranes were blocked for 1 hr at room temperature with 5% (w/v) skim milk powder in PBS with 0.1% Tween-20. PVDF membrane was incubated overnight at 4°C with primary antibodies diluted with PBS/Tween-20. The antibodies purchased from Santa Cruz BioTechnology, Inc. (California, USA) were: rabbit polyclonal IgG Bax (1:2500) (#sc-493), rabbit polyclonal IgG cyclin D1 (1:1000) (#sc-718), rabbit polyclonal IgG p21 (1:500) (#sc-56335), mouse polyclonal IgG p53 (1:500) (#sc-98), and mouse monoclonal IgG β-actin (1:2500) (#sc-1616). The rabbit polyclonal IgG NQO1 (1:2500) (#ab34173) was purchased from Abcam (Cambridge, MA, USA). The primary antibody was then removed and the blots were extensively washed with PBS/Tween-20. Blots were then incubated for 2 hr at room temperature with the secondary antibody horseradish peroxidase-labeled goat anti-mouse IgG (#sc-2005) or goat anti-rabbit IgG (#sc-2004) at 1:5000 dilutions in PBS. After removal of the secondary antibody and extensive washing in PBS/Tween-20, the blots were incubated in the ECL substrate solution (Amersham™ ECL™ prime Western Blotting detection reagent; GE Healthcare, Piscataway, NJ, USA). Densities of the specific bands of Bax, cyclin D1, p21, p53, NQO1 and β-actin were visualized and captured by ImageQuant™ LAS4000 (GE Healthcare).

### Statistical analysis

Data were expressed as mean ± SEM of triplicate assays from three independent experiments. An analysis of variance with repeated measurement was used to determine significant differences between each experimental group. The level of significance was set at *p* < 0.05.

## Results

### NQO1 expression in CCA cells is constitutively high and increased further by chemotherapeutic agents

**Figure 1:**
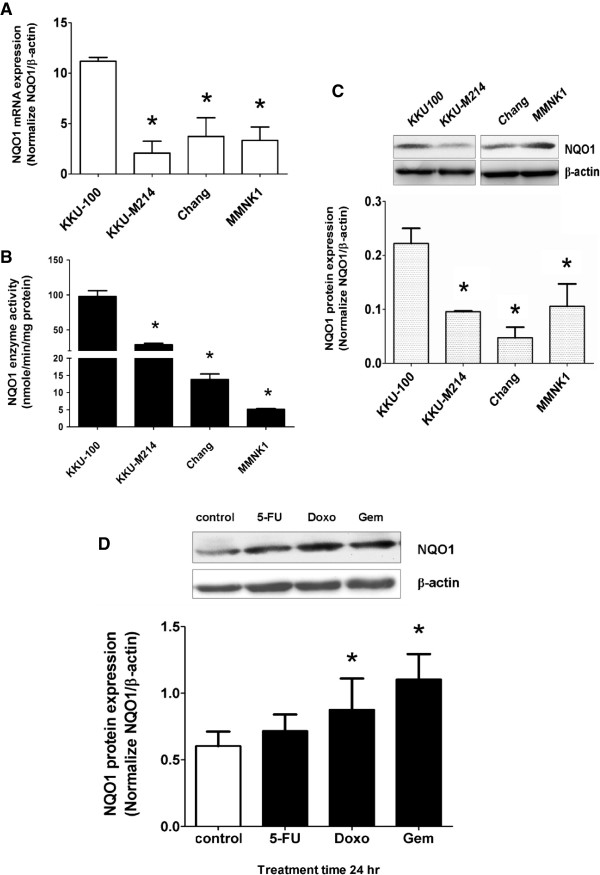
**Basal level of NQO1 mRNA, protein expression, and enzyme activity of CCA cells and NQO1 protein induction by chemotherapeutic agents (5-FU, Doxo, and Gem). (A)** Basal NQO1 mRNA expression in CCA cell lines (KKU-100 and KKU-M214) and two other cell lines (Chang and MMNK1 cells) analyzed by qPCR. The bars represent relative mRNA expression of NQO1 normalized with β-actin as internal control. *p < 0.05 vs KKU-100 cells. **(B)** Basal NQO1 enzyme activity analyzed by enzymatic methods. *p < 0.05 vs KKU-100 cells. **(C)** Basal NQO1 protein expression analyzed by Western Blot analysis using β-actin as internal control. Representative images of NQO1 and β-actin are shown in the top panel of the figure. *p < 0.05 vs KKU-100 cells. **(D)** Effect of chemotherapeutic agents on NQO1 protein expression in KKU-100 cells. Cells were exposed to 5-FU (3 μM), Doxo (0.1 μM), and Gem (0.1 μM) for 24 hr. Data represent mean ± SEM, each from three separated experiments. *p < 0.05 vs the untreated control.

We first examined the NQO1 expression in two CCA cell lines, KKU-100 and KKU-M214, and two other cell lines (liver Chang cells and bile duct epithelial MMNK1 cells). KKU-100 cells showed the highest expression in NQO1 mRNA, protein and enzymatic activity (Figure 1A-C). Chang and MMNK1 cell lines showed relatively low enzymatic activity. KKU-100 and KKU-M214 cells were used in the subsequent study as the representative of the high and low NQO1 expressing cells, respectively. To examine whether chemotherapeutic agents could induce the antioxidative stress response by induction of NQO1, KKU-100 was treated with 3 μM of 5-FU, 0.1 μM of Doxo, and 0.1 μM of Gem for 24 hr. The results showed that NQO1 protein expression was increased after treatment with Doxo and Gem, but not 5-FU (Figure 1D).

### NQO1 gene silencing sensitizes CCA cells to chemotherapeutic agents

**Figure 2:**
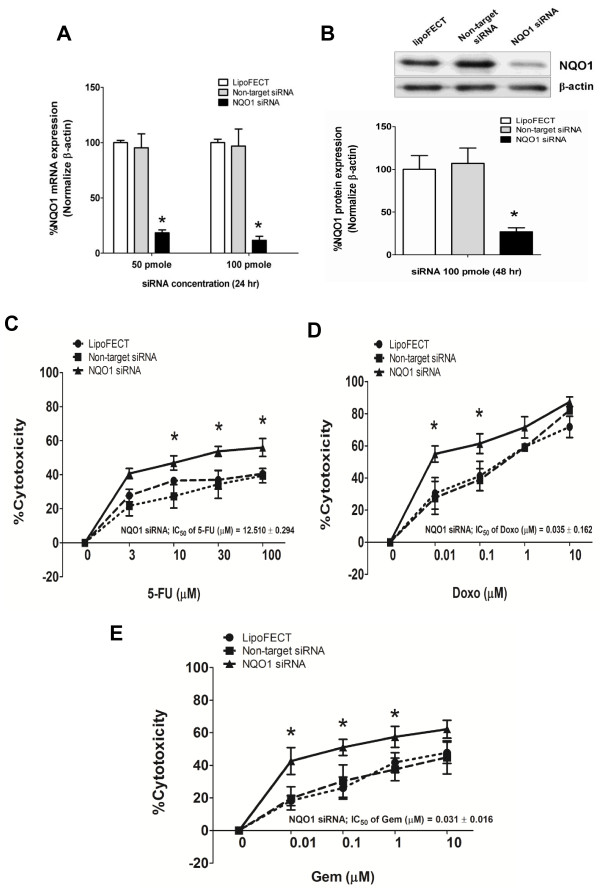
**Knockdown of NQO1 by siRNA sensitized KKU-100 cells to chemotherapeutic agents. (A-B)** Effect of NQO1 siRNA on mRNA and protein levels of NQO1 in KKU-100 cells. Cells were transfected with the pooled siRNA against NQO1 gene for 24 hr and 48 hr. Data represent mean ± SEM, each from three separated experiments. *p < 0.05 vs the non-targeting siRNA transfected cells. **(C-E)** Cytotoxicity of chemotherapeutic agents on NQO1 siRNA transfected KKU-100 cells. Forty-eight hour after transfection, cells were treated with varied concentration of chemotherapeutic agents; 5-FU, Doxo, and Gem for another 24 hr as described in the “Methods” section. The cytotoxicity was evaluated by SRB assay. Data represent mean ± SEM, each from three separated experiments. *p < 0.05 vs the non-targeting siRNA transfected cells.

To verify the possibility that NQO1 can modulate the susceptibility of CCA cells to chemotherapeutic agents, NQO1 expression was knocked down by using a siRNA method. KKU-100 cells were used in the study, because the recent study has shown that the high NQO1 expressing cells, KKU-100 cells, are sensitized by dicoumarol to the cytotoxicity of chemotherapeutic agents, while the low expressing cells are not [22]. The results showed that NQO1 mRNA expression was suppressed by siRNA more than 80% at 24 hr (Figure 2A). The protein expression levels (Figure 2B) and enzymatic activity (data not shown) were also suppressed moderately at 24 hr (data not shown) and about 80% at 48 hr after the siRNA transfection. The further experiment was performed after transfection for 48 hr.

Then, we examined the susceptibility of NQO1-knockdown-KKU-100 cells to various chemotherapeutic agents. NQO1 siRNA treatment alone did not alter significantly the cell viability compared with that of KKU-100 cells treated with non-target siRNA. By NQO1-knockdown, KKU-100 cells became more sensitive to the cytotoxic effect of 5-FU, Doxo, and Gem (Figure 2C-E). The chemosensitizing effect was remarkable especially at the low concentrations of the chemotherapeutic agents.

### NQO1-knockdown and chemotherapeutic agent treatment induce p53 and altered expression of cell death pathway proteins

**Figure 3:**
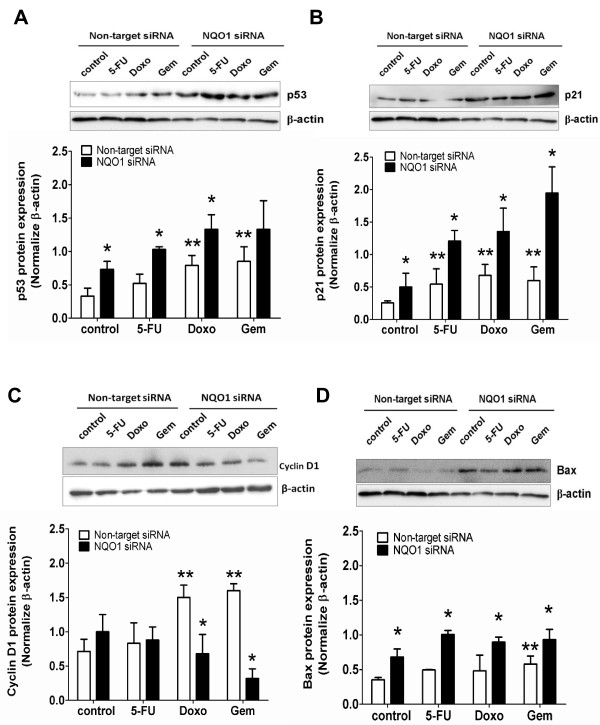
**Altered expressions of proteins related to cell proliferation and apoptosis pathways. A-D**, Expressions of proteins related to cell proliferation and apoptosis pathways. KKU-100 with NQO1 knocked down cells were exposed to chemotherapeutic agents; 5-FU (3 μM), Doxo (0.1 μM), and Gem (0.1 μM) for 24 hr. Whole cell lysates were prepared after indicated treatment and Western blot analysis was conducted using anti-p53 **(A)**, -p21 **(B)**, -cyclin D1 **(C)**, -Bax **(D)** and -β-actin antibodies. The relative bars that were normalized with β-actin as a loading control of each band is shown below the Western blot images. Data represent mean ± SEM, each from three separated experiments. *p < 0.05 vs the treated non-targeting knocked down cells. **p < 0.05 vs the untreated non-targeting knocked down cells.

To explore the possible mechanisms of chemosensitizing effect of NQO1-knockdown, we examined the expression levels of cell death-related proteins in NQO1-knockdown-KKU-100 cells. Western blot analyses revealed that Doxo and Gem treatment alone increased p53 levels (Figure 3A). When NQO1-knockdown-KKU-100 cells were treated with chemotherapeutic agents, p53 level was enhanced further by all 3 agents (Figure 3A). Then, we examined the expression levels of some p53 downstream proteins, i.e. p21, cyclin D1, and Bax protein. Similar to p53, p21 and Bax were over-expressed by the drug treatments (Figure 3B, 3D). In contrast, in the NQO1 knockdown cells, treatment with chemotherapeutic agents strongly suppressed the cyclin D1 level (Figure 3C). In the non-target siRNA transfected KKU-100 cells, Doxo and Gem, but not 5-FU, treatments increased cyclin D1 expression (Figure 3C).

### Over-expression of NQO1 in CCA cells induces drug resistance against chemotherapeutic agents

**Figure 4:**
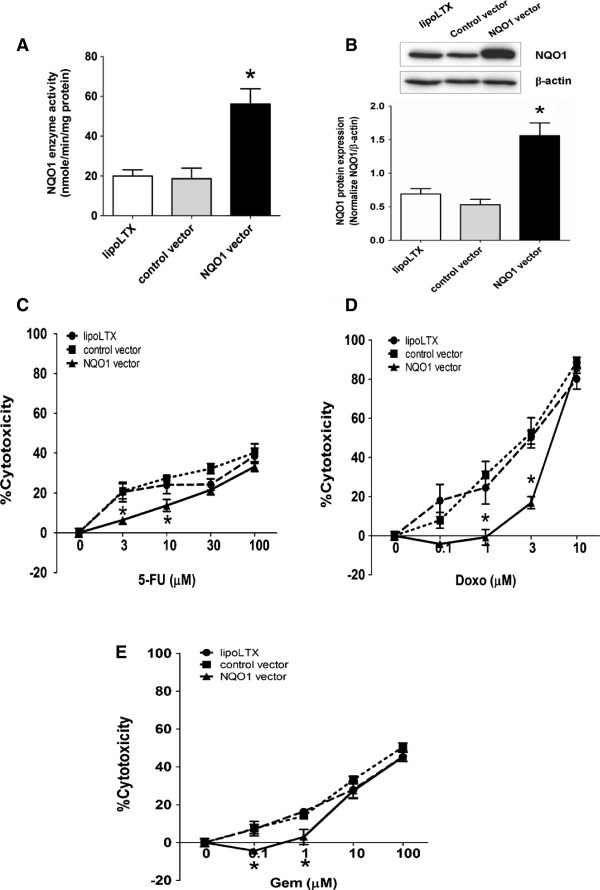
**Effects of NQO1 over-expression on the susceptibility of KKU-M214 cells to chemotherapeutic agents (5-FU, Doxo, and Gem). A-B**, Effect of NQO1 over-expression on mRNA and protein levels of NQO1 in KKU-M214 cells. The pCMV6-XL5-NQO1 (wild type NQO1) or pCMV6-XL5 (control vector) was transfected to KKU-M214 for 24 hr. The whole cells were collected for NQO1 enzyme activity assay **(A)** and Western blot analysis **(B)**. The data represent mean ± SEM, each from three experiments. *p < 0.05 vs the control vector transfected cells. **(C-E)** Cytotoxicity of chemotherapeutic agents on NQO1 over-expressed KKU-M214 cells. Twenty-four hour after transfection, cells were incubated with chemotherapeutic agents for additional 24 hr (Doxo) and 48 hr (5-FU and Gem). The cytotoxicity was evaluated by SRB assay. Data represent mean ± SEM, each from three separated experiments. *p < 0.05 vs the control vector transfected cells.

Since KKU-M214 cells naturally express relatively low level of NQO1, effects of NQO1 over-expression by transient transfection with NQO1 expression vector on the susceptibility of cells to chemotherapeutic agents was evaluated. After transfection, the NQO1 enzyme activity in the transfected cells was elevated approximately 2.5-fold and the NQO1 protein level was 2.25-fold higher than the control vector (Figure 4A-B), indicating that NQO1 construct was efficiently expressed in KKU-M214 cells. Then, NQO1-over-expressed KKU-M214 cells were exposed to 5-FU and Gem for 48 hr, and to Doxo for 24 hr. The results showed that the cytotoxicity of 5-FU, Doxo, and Gem were markedly decreased for NQO1-over-expressed KKU-M214 cells (Figure 4C-E), indicating the protective effect of NQO1.

### Over-expression of NQO1 suppresses chemotherapeutic agents-induced p53 and protein expression in the cell death pathway

**Figure 5:**
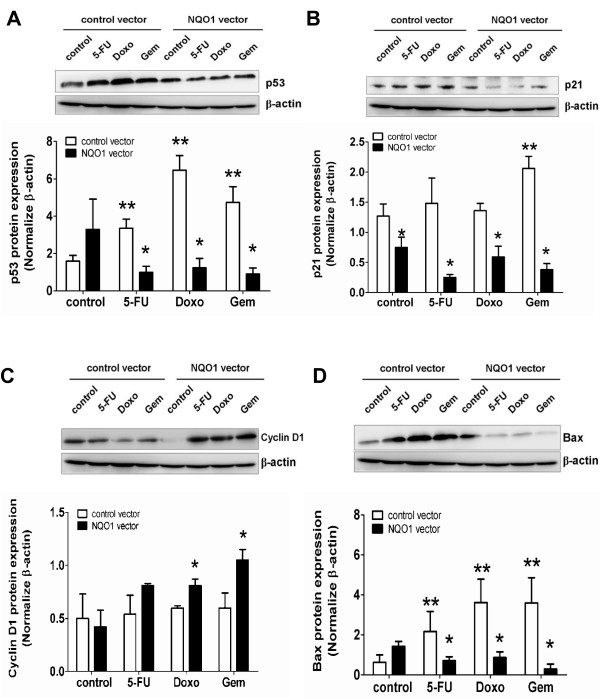
**NQO1 over-expression attenuates the p53 pathway in KKU-M214 cells. A-D**, Western blots of p53 **(A)**, p21 **(B)**, cyclin D1 **(C)**, and Bax **(D)** protein in KKU-M214-NQO1 over-expressed cells after treatment with 5-FU 3 μM (48 hr), Doxo 0.1 μM (24 hr), and Gem 0.1 μM (48 hr). The relative bars that were normalized with β-actin of each band are shown below the Western blot images. *p < 0.05 vs the treated control vector transfected cells. **p < 0.05 vs the untreated control vector transfected cells.

Previous experiment showed that NQO1-knockdown increased p53 and apoptogenic protein expression. The results of this experiment showed that over-expression of NQO1 in KKU-M214 cells strongly suppressed the chemotherapeutic agents-induced increased expression of p53, p21, and Bax (Figure 5A-B & D). On the other hand, over-expression of NQO1 enhanced Doxo- and Gem-induced cyclin D1 expression (Figure 5C).

### Knockdown of p53 abolishes the chemosensitizing effect of NQO1 silencing

**Figure 6:**
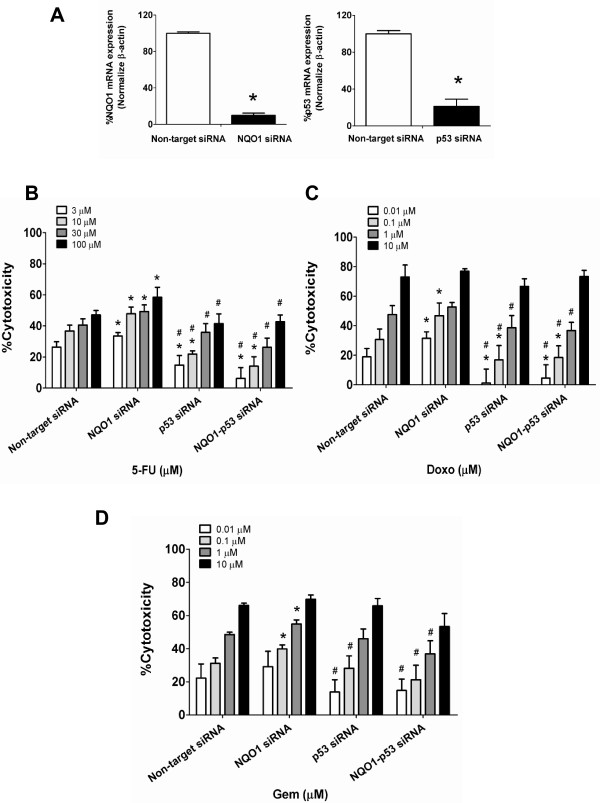
**Double knockdown of NQO1 and p53 by siRNA altered KKU-100 cells to chemotherapeutic agents. (A)** Effect of co-transfected NQO1 and p53 siRNA in KKU-100 cells. Cells were transfected with the pooled siRNA against NQO1 and p53 for 24 hr. The bars represent relative expression of NQO1 and p53 normalized with β-actin as internal control. **(B-D)** After co-transfection, cells were treated with varied concentrations of chemotherapeutic agents; 5-FU, Doxo, and Gem for another 24 hr as described in the “Methods” section. The cytotoxicity was evaluated by SRB assay. Data represent mean ± SEM, each from three separated experiments. *p < 0.05 vs the non-targeting knocked down cells and ^#^*p* < 0.05 vs NQO1 knocked down cells.

Since the results given above showed that the knockdown and over-expression of NQO1 enhanced and suppressed, respectively, the chemotherapeutic agent-mediated cytotoxicity in association with the altered expression of p53, p53 apparently play a role in the expression of the cytotoxic effect of those anti-cancer agents. To validate the role of p53, we prepared the double knockdown of NQO1 and p53 in KKU-100 cells. The efficiency of NQO1 and p53 knockdown was more than 80% (Figure 6A). As is shown above, NQO1-knockdown increased the susceptibility of KKU-100 cells to chemotherapeutic agents. Conversely, p53-knockdown markedly reduced cytotoxic effect of all tested chemotherapeutic agents compared with chemotherapeutic agents alone (Figure 6B-D). Interestingly, in the double knockdown experiment, the cytotoxic potentiation effect of NQO1 gene silencing was totally diminished by the simultaneous knockdown of p53. The cytotoxic effects of chemotherapeutic agents on double knockdown cells were similar to those on p53 knockdown cells. These results strongly suggest that the cytotoxic effects of all 3 chemotherapeutic agents on CCA cells were dependent on p53 expression and NQO1 is probably the upstream modulator of p53.

## Discussion

We previously showed that the survival time of CCA patients with high NQO1 mRNA expression was shorter than patients having CCA with low NQO1 expression [21], suggesting the possible role of NQO1 in CCA progression. We also demonstrated that inhibition of NQO1 in high NQO1 expressing cell line, KKU-100, enhanced the cytotoxic effect of chemotherapeutic agents, but not in the low NQO1 expressing cells, i.e. KKU-M214 [22]. In the present study, the role of NQO1 was validated by knockdown of NQO1 expression in KKU-100 cells and over-expression of NQO1 in KKU-M214 cells. Knockdown of NQO1 enhanced the cytotoxic effect of 5-FU, Doxo and Gem, whereas over-expression of NQO1 protected the cells from chemotherapeutic agents. The suppression of NQO1 expression was associated with up-regulation of p53, p21, and Bax proteins, while over-expression was associated with down-regulation of those proteins. The role of NQO1 in cell viability became significant when NQO1 knockdown KKU-100 cells exposed to chemotherapeutic agents. It should be noted that NQO1 plays an important role in cell viability especially at severe stress condition in CCA cells. The role of p53 was verified by p53 and NQO1 gene silencing with siRNA. The potentiation effect of NQO1 gene silencing on the cytotoxicity of chemotherapeutic agents was inhibited by p53 knockdown. Thus, the sensitizing effect of NQO1 is likely to be mediated via p53.

Inhibition of NQO1 by dicoumarol suppressed cancer cell growth and potentiated the cytotoxicity of chemotherapeutic agents [19,20]. Chemotherapeutic agents such as Doxo and Gem induced over-expression of NQO1 in CCA cells. This may be a cellular adaptive response to oxidative stress and cytotoxicity [13] and may confer the cytoprotective effect to the cells. The biological role of NQO1 in CCA was validated in this study and found to be consistent with our recent report in that suppression of NQO1 enhances the cytotoxic effect of many chemotherapeutic agents and the activation of mitochondrial death pathway [22]. On the other hand, over-expression of NQO1 in KKU-M214 cells suppressed the cytotoxic effect of chemotherapeutic agents. The results indicated the protective effect of NQO1 from chemotherapy in CCA. Taken together, this may provide a possibility to combine NQO1 inhibitor together with chemotherapy as a novel treatment strategy for CCA. However, to apply this information to CCA patients, several critical studies are requested to confirm the *in vivo* relevance of these findings. For example, the synergistic role of NQO1 inhibition in chemotherapy of CCA should be further validated in animal models. This could be carried out in our future study.

The mechanism of NQO1-mediated chemosensitization was further explored. Previous reports suggested that NQO1 modulates p53 expression by interfering with 20S proteasome-mediated degradation of p53 [24]. Inhibition of NQO1 by dicoumarol suppressed p53 protein levels and induced cell death [24]. In contrast, dicoumarol at non-cytotoxic concentrations, but sufficient to inhibit NQO1 enzyme activity, enhanced p53 protein levels [22]. Present results show that the suppression of NQO1 increased p53 expression.

Tumor protein p53 and Bcl family proteins regulate mitochondrial outer membrane permeabilization (MOMP) [26]. Our results showed that the increase of p53 was associated with increased p21 and Bax levels. Both p21 and Bax are p53-dependent downstream gene products. The p21 is a potent cyclin-dependent kinase inhibitor and its expression is associated with the strong antiproliferative effect as was seen in the present study. Bax is a multidomain proapoptotic Bcl2 family. It translocates into the mitochondrial outer membrane and forms Bax pores leading to the release of proapoptotic proteins and ensuing cell death [27]. p53 is a tumor suppressor gene that responded to DNA damage or oxidative stress by inducing growth arrest or apoptotic cell death [28,29]. Our results showed that knockdown of p53 inhibited the chemosensitizing effect, which was induced by knockdown of NQO1 in KKU-100 cells. This indicates that the sensitizing effect of NQO1 knockdown is mediated via p53 pathway. It is also noted that KKU-100 cells expressed both the wild type full length p53 as well as the splicing variant of truncated p53 protein [30]. Interestingly, our results showed that the potentiation effect of NQO1 gene silencing on the cytotoxicity of chemotherapeutic agents can occur even in cancer cells with high expression ratio of mutant p53/wild type p53. It is yet to determine the chemosensitizing effect of NQO1 suppression on cells expressing the other mutated p53. As some CCA patients express high NQO1 [20], targeting the NQO1 by suppressing the activity or expression could be a strategy to overcome drug resistance of cancer and enhancing the efficacy of chemotherapeutic agents.

## Conclusions

In summary, NQO1 plays an important role in cytoprotection of cancer cells and modulates the sensitivity of chemotherapeutic agents, particularly in the high NQO1 expressing CCA cells. NQO1 is a potential molecular target for enhancing the antitumor activity of chemotherapeutic agents.

## Abbreviations

NQO1: NAD(P)H-quinone oxidoreductase 1; CCA: Cholangiocarcinoma; 5-FU: 5-fluorouracil; Doxo: Doxorubicin; Gem: Gemcitabine; siRNA: Small interfering RNA; SRB: Sulforhodamine B; p53: Tumor protein 53.

## Competing interests

The authors declare that they have no competing interests.

## Authors’ contributions

Conceived and designed: PZ AP VK. Performed the experiments: PZ AP LS BS. Analyzed the data: PZ AP VK. Wrote the paper: PZ AP VK. All authors read and approved the final manuscript.
